# Does workplace social capital predict care quality through job satisfaction and stress at the clinic? A prospective study

**DOI:** 10.1186/s12889-021-11320-8

**Published:** 2021-07-05

**Authors:** Hanne Berthelsen, Mikaela Owen, Hugo Westerlund

**Affiliations:** 1 Centre for Work Life and Evaluation Studies (CTA) & Faculty of Odontology, Malmö University, Malmö, Sweden; 2 Centre for Workplace Excellence, University of South Australia, Adelaide, SA Australia; 3 Department of Psychology, The Stress Research Institute, Stockholm University, Stockholm, Sweden

**Keywords:** COPSOQ, Psychosocial work environment, Performance, Dental fillings, Dentistry, Health care

## Abstract

**Background:**

Welfare societies like Sweden face challenges in balancing the budget while meeting the demand for good quality healthcare. The aim of this study was to analyse whether care quality, operationalized as survival of dental fillings, is predicted by workplace social capital and if this effect is direct or indirect (through stress and/or job satisfaction among staff at the clinic), controlling for patient demographics.

**Methods:**

The prospective design includes A) work environment data from surveys of 75 general public dental clinics (aggregated data based on 872 individual ratings), and B) register-based survival of 9381dental fillings performed during a 3-month period around the time of the survey, and C) patient demographics (age, gender, income level and birth place). Using a multi-level discrete-time proportional hazard model, we tested whether clinic-level social capital, stress, and job satisfaction could predict tooth-level filling failure, controlling for patient demographics. One direct and two indirect pathways, moderated by filling tooth, location, and filling type, were tested.

**Results:**

High workplace social capital reduced the risk of early failure of fillings in molar teeth, mediated by group-perceived job satisfaction (indirect path: OR = 0.93, *p* < .05, direct path from job satisfaction: OR = 0.89, *p* < .05). Contrary to expectations, we found no support for a direct effect from social capital on care quality or for the indirect pathway via stress at the clinic level.

**Conclusions:**

Workplace social capital boosted the quality of dental fillings through increased levels of job satisfaction. In addition, staff at clinics with higher social capital reported less stress and higher levels of job satisfaction. These results indicate that promotion of social capital may improve both occupational health and care quality.

## Introduction

The cost of healthcare in Sweden has increased from 5.5% of GNP in 1970 to 11.0% in 2019 [1], which illustrates the major challenges in welfare societies of balancing the budget while meeting the demand for high quality healthcare [2, 3]. In the US, a framework for the improvement of healthcare (the Triple Aim Principle) was adopted 2008 and has been widely implemented as part of the national healthcare strategy. The primary aim of the framework is that healthcare institutions will work to improve public health through simultaneously enhancing patient experience and reducing per capita cost [2]. In order to build high-quality healthcare services, however, attention also needs to be directed to the work environment and occupational health of the healthcare providers, i.e., the Triple Aim can be expanded to the Quadruple Aim Principle [4]. In line with these strategies for healthcare improvement, the Swedish government established the “Delegation for Trust-Based Public Management” in 2016 with the goal of enhancing care quality by promoting more trust-based governance and management of welfare services. This idea of trust in relationships is a central component across various operationalizations of workplace social capital, particular in the Nordic countries (i.e., [5–9]). Oksanen et al. distinguish between a vertical dimension (e.g., relationships between managers and employees) and a horizontal dimension (e.g., relationships among employees at same hierarchical level) of workplace social capital [9], while Clausen et al. operate with four dimensions (linking (between managers and employees), bridging (between different work teams), bonding (within a work team) and overall social capital) [8]. In the third version of Copenhagen Psychosocial Questionnaire, COPSOQ III, however, workplace social capital is operationalized as a collaborative work climate characterized by mutual trust (vertical and horizontal) and justice [6, 7].

Social capital in the workplace predicts motivational and health-related outcomes, e.g. job satisfaction, work engagement, sickness absence, workability, and psychological wellbeing [10–18], as well as self-rated performance [8, 19]. The work environment can affect quality of care either indirectly (that is, via factors like stress and job satisfaction that may affect professional behaviour) or directly (that is, the work environment or staffing issues can promote systematic errors or compromise quality) [20]. In the present study we will investigate the relationships between workplace social capital, job satisfaction, and stress and the impact on longevity of dental fillings, a register-based indicator of care quality in the context of dentistry.

To understand how social capital in the workplace works in dentistry, it is important to understand how dentistry is typically organized. In familiar contexts like hospital work, during a typical work week, any particular staff member might work in different teams (i.e. operating room, outpatient clinic, and ward) in their department, and the work can take place at any time of day or night in different shifts. This mobility makes it difficult to clearly define small, separate work teams in the hospital setting. In contrast, the organization of dentistry is less complex. Dentistry has well-defined, geographically separated and relatively small, locally managed workplaces and access to registers that include objective measures of care quality. Having small, restricted teams makes dentistry a suitable setting for studies on workplace social capital. Typically, the local leader of clinics has an educational background in dentistry and takes part in clinical work. The unidimensional measure of workplace social capital suggested by COPSOQ III [6, 7] fits well to the context of dentistry, because there is a small power distance between employees and the local manager in clinics, and these clinics are mutually independent.

Throughout Europe, healthcare workers (more often than workers in other sectors) report insufficient time for task completion, frequent interruptions, exposure to angry clients, emotionally disturbing situations, and a need to hide their emotions (p 50 in [21]). An intense work environment is also characteristic for dentistry workplaces, because demands are high and many employees experience high levels of work-related stress [22, 23]. In various healthcare settings, links have been repeatedly made between poor wellbeing outcomes for workers and poor patient safety outcomes (for review, see [24]), but to date, little research has investigated the link between worker stress and care quality in dentistry [25].

Dental caries (also known as tooth decay or cavities) is the most common oral disease and a significant segment of the global burden of disease [26]. The standard treatment for dental caries is dental fillings, but despite extensive technical research into filling materials, the longevity of fillings is still a matter of concern [27]. A review of the findings of long-term clinical studies concluded that the main problem of filling failure goes beyond the materials – the issues also extend to factors related to the care providers and patients [28]. Dental filling is a complex task involving many steps that require careful attention if the optimal technical properties are to be reached. If there is insufficient time for staff to complete all tasks accurately, these properties will not be reached and the filling might eventually need replacement [28]. Collaboration with the patient can also become complicated because stress from the patient can spill over to the dental team and vice versa [29]. Dentists in high-pressure workplaces report that their clinical decision making is sometimes compromised [30, 31], a finding consistent with a small-scale experimental study in which time pressure reduced the quality of dentists’ diagnostic performances [32]. We have found no previous studies investigating the influence of perceived stress, as experienced at the clinic level, on the quality of dental fillings. Therefore, the present study will address this gap and explore the association between workplace stress and dental filling failure. We anticipate that high stress reported by an entire staff-group will implicate an increased risk of early failures of dental fillings, especially for fillings requiring the most complicated work procedures.

While dentistry can be emotionally demanding and often stressful, the job also confers feelings of meaningfulness, professional pride, engagement, and job satisfaction, especially in working conditions that support opportunities for providing high quality care [33, 34]. The Happy Worker Hypothesis, which has its roots in the Hawthorne studies from the beginning of the last century [35], claims that satisfied workers perform better and are more productive [36]. Today, this hypothesis is central to the positive organisational psychology movement and is considered to be the ‘Holy Grail’ of management research [37]. Frederickson’s broaden-and-build framework [38] can be used to explain this Happy Worker hypothesis: When employees are satisfied and psychologically well, it is more likely that they will have the resources necessary for better job performance [37]. It has repeatedly been found that job satisfaction and work engagement of the individual care provider lead to higher productivity and better quality (for an overview see e.g. [36]) and that work engagement is associated with dentist productivity [39]. Previous studies have mainly addressed these issues at the individual level [40], but Taris and Schreurs [41] found partial support for the model also at the organizational level. Gain spirals can arise from job resources to engagement and work-unit innovation, e.g. constantly making improvements at the dental clinic [42]. In line with this, we hypothesize that overall job satisfaction level at the clinic will be associated with a reduced risk of early failures of dental fillings, in particularly for fillings requiring complicated work procedures.

Finally, workplace social capital could also be important for the longevity of dental fillings, because dentistry relies on teams composed of staff members with different occupational backgrounds that communicate, collaborate and coordinate many tasks in relation to patients during a workday. Dentists consider interpersonal trust-based relationships and good communication to be fundamental for the team to provide high quality dental care [33]. Previous research has shown that social capital leads to a higher engagement in clinical improvements [12], more helping behaviours [43], and can be a buffer in emotionally demanding situations [44]. Further, the organisational justice climate at dental clinics is of importance for how staff members rate the care quality [45]. For these reasons, we expect workplace social capital will correspond to an increased longevity of dental fillings made at the clinic, and for there to be an indirect effect through job satisfaction and stress among the staff.

### Aim/research question

The overall objective is to investigate the relationship between work environment factors at the clinic level and dental care quality. More concretely, we will analyse whether longevity of dental fillings are linked with workplace social capital and whether any indirect effect can be measured by the average level of stress or job satisfaction among staff at the clinic, controlling for the potential confounding effects of patient demographic factors.

Specifically, we hypothesize that:

1. Higher levels of workplace social capital will be associated with higher average levels of job satisfaction and lower average levels of stress at the clinics.
2. Higher levels of job satisfaction at clinics lead to a lower risk of patients experiencing early filling failure.
3. Higher levels of stress at the clinics lead to a higher risk of patients experiencing early filling failure.
4. Higher levels of workplace social capital will indirectly (via average levels of job satisfaction and stress at the clinic) lead to a lower chance of early filling failure.
5. Characteristics of the dental filling (filling tooth, location, and filling type) will moderate the potential effect of work environment on the risk of early filling failure. We expect that the more complicated the filling procedure is due to local conditions in the mouth, the more the risk of an early failure of the filling will be influenced by work environment.

## Methods

This study has a prospective design including baseline work environment data from surveys on dental clinics and register-based follow-up of dental care quality and patient demographics.

### Participants and materials

Questionnaire data on the work environment was collected 2014–2015 among staff at all public dental practices in four regions of Sweden. Inclusion criteria for the present study were general dental practices, a minimum of 2 practitioners at the clinic, and a minimum of 3 staff members in total who had responded to the work environment survey. Data from 75 general public dental clinics, based on 872 individual ratings from non-managerial employees, are included in the analyses and with a response rate of 73% after two reminders.

*Social capital* at the clinic was operationalised by the COPSOQ III [6, 7, 46] by 6 items addressing trust in relationships and organisational justice rated on a 5-point Likert scale. An additive index scale was constructed and converted to a metric score range 0–100 [7]. For each clinic, an average scale score was calculated. *Stress* (4 items) and *Job satisfaction* (4 items) by COPSOQ II [47, 48] were rated on 5-point Likert scales. Additive index scales ranging from 0 to 100 were constructed, setting the scale scores as missing if respondents had answered fewer than two items on a scale and aggregated to clinic scores.

Outcome data on dental care quality, operationalized as *survival of dental fillings*, were obtained from the Swedish Quality Registry for Caries and Periodontal Disease, SKaPa. For patients aged 20 or older, all class 2 and 3 composite fillings in premolars and molars that were made at the clinics across 3 months; a month prior, the month of, and the month following the work environment survey, and then also followed for up to 4 years. The analyses of how the work environment affects care quality could be confounded by systematic variation among clinics in regard to patient characteristics, so for that reason, *demographic background* data on the age, gender, income level and place of birth of the patients were obtained from Statistics Sweden. As suggested by a 13-year observation study, *moderating local factors* could be filling tooth (molar or premolar), location (lower jaw or upper jaw), or filling type (3 or 2 surfaces included in the filling) [49].

### Analysis

**Fig. 1:**
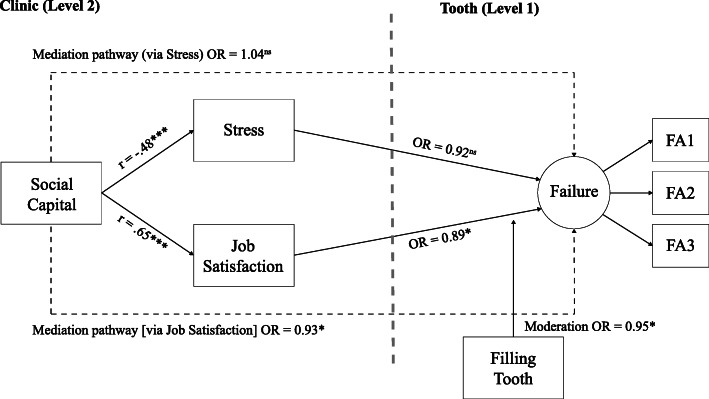
The findings for the proposed model from work environment at the clinic level (*n* = 75) to patients filling failure (*n* = 9381) with indirect paths to failure through stress and job satisfaction with filling tooth moderation, controlling for age, gender, education level, income, and country of birth. Note: r = linear regression, OR = odds ratio

Using a multi-level discrete-time proportional hazard model analysis with moderation in Mplus v8, we tested the effect of work environment on filling failure (see Fig. 1). Specifically, we tested the direct and indirect effects of clinic-level (level 2) social capital, stress, and job satisfaction on the probability of experiencing a filling failure at the tooth level (level 1), controlling for patient demographics (e.g., patient age, gender (male), education (three levels), income (strata of 100,000 SEK per year), and country of birth (Scandinavia/other)). The two indirect pathways were from social capital to filling failure through stress and through job satisfaction. These indirect pathways also included moderation. We tested the moderated pathways from job satisfaction to filling failure, and stress to filling failure, by filling tooth, location, and filling type. Moderation was tested by creating interactions with the slopes command in the ‘Model’ section of analysis.

Work environment factors (social capital, stress, and job satisfaction) were clustered at the clinic level (Level 2, see Table 1 for intraclass co-efficient (ICC) scores), and filling failure was at the tooth level (Level 1). To justify aggregating the individual-level scales scores for social capital, stress and job satisfaction to an average clinic score, the ICC(1) and ICC(2) were calculated along with r_wg(j)_. ICC(1) represents the amount of variance in the employees’ responses that can be explained by the clinic they work in [50–53]; in applied field research of organisations, these values are typically up to a maximum of 0.20 (p 362 in [51]. ICC(1) values of 0.05 can be considered a small to medium effect while higher values indicate stronger effects [53]. ICC(2) is an estimate of the reliability of the aggregated group means [50–52]. Values < 0.5 indicate poor reliability, 0.50–0.75 indicate moderate reliability and > 0.75 indicate good reliability of group-level means [54]. Finally, r_wg(j)_ reflects the consistency in response within the groups, that is, the extent to which there is within-group agreement in the workplace [51], with values of 0.70 or above generally considered to be acceptably consistent with other measures of reliability [55]. Overall, the ICC scores and r_wg(j)_ scores indicate whether it is appropriate to aggregate the individual level scales. While values for job satisfaction and stress fell just shy of the 0.50 recommendation for ICC(2) values, there were reasonable values for ICC(1) and r_wg(j)_ (Table 1).

**Table 1:** Intraclass Correlation Coefficients (ICC) and scale characteristics for the three work environment measures

	M	SD	ICC(1)	ICC(2)	r_wg(j)_	Cronbach’s alpha
Social Capital	67.71	9.21	0.25	0.79	0.93	0.86
Social Capital (metric)^a^	45.48	8.94	0.20	0.75	n/a	n/a
Stress	37.44	10.44	0.07	0.47	0.70	0.91
Job Satisfaction	65.92	7.54	0.07	0.47	0.85	0.83

Note. ^a^the variable used in analysis

All three work environment factors were entered as observed variables in the model, and failure of filling was constructed as a latent variable. The filling failure latent variable comprised our three binary event indicators (year 1, year 2, and year 3) which corresponded to three time periods. If our event of interest (filling failure) occurred, scores were coded as 1; if our event of interest did not occur (no filling failure), scores were coded as 0. Once the event of interest occurred, the remaining event indicators for the subsequent time periods were coded as missing. The latent loadings for filling failure were constrained to 1 (consistent with a proportional hazard model), and the variance of filling failure variance was constrained to 0.

Using Maximum Likelihood Restricted (MLR) estimation consistent with proportional hazard models, we tested the linear regression pathways from social capital to stress and to job satisfaction, and the logit regression pathways from social capital, stress, and job satisfaction onto filling failure. Social capital, stress, and job satisfaction were all standardized via the DEFINE command in Mplus prior to analysis to provide standardized coefficients for both the linear regression and logit regression pathways. Finally, all logit regression coefficients were converted to Odds Ratio coefficients.

Two models testing the pathways from workplace social capital to filling failure via stress and job satisfaction, moderated by filling tooth, filling location and filling type, were tested. If we were to follow the causal method of mediation outlined by Baron and Kenny [56], a direct path from the predictor to the outcome would need to be established. Of note, the causal approach has recently drawn criticism due to its lack of power and statistical parsimony through the increased number of null hypotheses requiring rejection and consequent increased chances of making a type 2 error [57]. Additionally, the causal approach can prematurely reject an indirect effect if there is a mediator with a positive impact (e.g., stress) and a mediator with a negative impact (e.g., job satisfaction) that cancel each other out in the direct effect [57]. For these reasons, consistent with Hayes [57], our indirect effect does not require an association between the predictor variable (social capital) and the outcome variable (filling failure probability) [58].

Model 1 included the indirect paths from social capital at the clinic level (Level 2) to filling failure at the tooth level (Level 1) via job satisfaction and stress while controlling for patient demographic information and correlating stress and job satisfaction error terms. The pathways from social capital, job satisfaction, and stress were moderated by filling type, size, and location in the mouth. In Model 2, a direct pathway from social capital to filling failure was added with all else held consistent. To measure model fit we used three fit indices, namely 2*Loglikelihood (Deviance), the Akaike Information Criteria (AIC), and sample-size adjusted Bayesian Information Criteria (AdjBIC). For all fit indices, lower scores indicate better model fit. To compare the fit between models, we used the Satorra-Bentler scaled chi-square test (TRd) [59], which uses the deviance scores and correction factors across the two comparison models to detect significant differences [60].

## Results

Over the course of this study, a total of 8505 patients received 9997 dental fillings fulfilling the inclusion criteria, and we were able to include 8278 of these in the study (1719 fillings were omitted because due to missing data for filling longevity). Most patients received either one (85.9%) or two fillings (11.5%). Fillings in molar teeth were most frequent (61.8%), and among these, 20.8% needed a replacement during the observation period.

The average age of patients was 50.2 years (SD 15.7), 50.1% were women, 90.5% were born in a Scandinavian country, and the average total yearly income was SEK 296,161 (SD 161,276) (100 SEK was equivalent to 9.1 USD on 2 January 2017). The highest education levels of the patients were 28.9% at the primary level, 37.9% at the secondary level and 27.1% had some higher education; the educational level was unknown for 6.1%.

The tests of Model 1 showed, as expected, that social capital was positively associated with job satisfaction and negatively associated with stress (cf. Figure 1). In this model, on the pathway from social capital to filling failure through job satisfaction and stress, it was found that increases in job satisfaction were associated with a decreased risk of filling failure (OR = 0.89, 95%CI [0.81, 0.99], *p* < .05). Additionally, the indirect path from social capital to filling failure through job satisfaction was significant (OR = 0.93, 95%CI [0.87, 0.998], *p* < .05). No path was found from stress to filling failure. In Model 2, a direct path from workplace social capital to filling failure was added. However, the direct path from social capital to filling failure was not significant. Overall, using the Satorra-Bentler scaled chi-squared test, no significant difference was found between the two models (TRd (1) = 0.62, *p* = 0.431,) indicating that the addition of a direct pathway did not improve model fit (Table 2).

**Table 2:** Model fit and comparison of work environment to filling failure in dental clinics

	Deviance	Correction Factor^a^	Free Parameters	AIC	AdjBIC
Model 1	9587.72	1.20	20	9627.72	9707.09
Model 2	9586.64	1.23	21	9628.64	9711.98

*Note. AIC* Akaike Information Criteria, *AdjBIC* Sample-Size Adjusted Bayesian Information Criteria. ^a^Used for the Satorra-Bentler test

The only significant moderation on the association between job satisfaction and filling failure was of type of tooth getting the filling (OR = 0.95, 95%CI [0.90, 0.99], *p* < .05). For molar teeth, this association was statistically significant (OR = 0.88, 95%CI [0.80, 0.96], *p* < .05), but not for premolar teeth (OR = 0.93, 95%CI [0.80, 1.06], *p* = .405). No significant moderation was found for filling type or filling location for the pathways from stress and job satisfaction onto filling failure, and filling tooth on the pathway between stress and filling failure (*p* > .05).

**Fig. 2:**
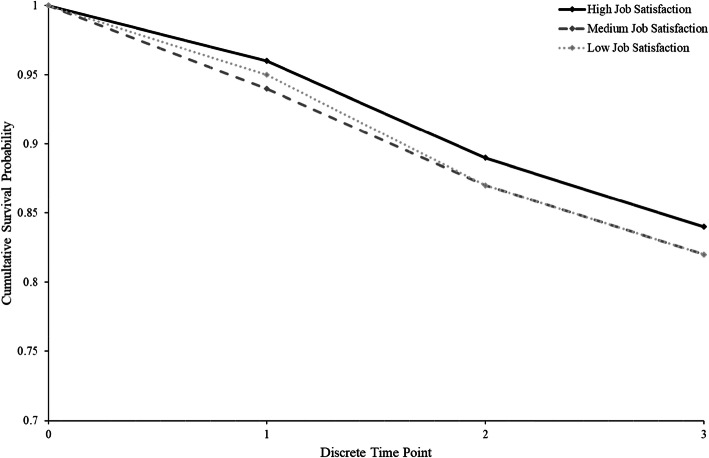
Cumulative survival probabilities of fillings for molar teeth based on clinic-level assessments of job satisfaction. Note: low job satisfaction is 2 SD below the mean, medium job satisfaction is the mean level job satisfaction, and high job satisfaction is 2 SD above the mean

Figure 2 presents the cumulative survival probability for fillings on molars, as a function of clinic-level rated job satisfaction. With low and medium levels of clinic-level job satisfaction, the cumulative survival probability drops at a faster rate than it does for high job satisfaction. Clinics with high levels of staff job satisfaction had consistently higher levels of survival probability across the three discrete time periods.

## Discussion

This study is the first to investigate whether work environment factors can predict dental care quality. We investigated the relationships among workplace social capital, job satisfaction, and stress at dental clinics, and the impact that these factors have on a register-based indicator of care quality. Our main finding was that increases in workplace social capital reduced the risk of early failure of fillings in molar teeth, and that this effect was indirect, taking effect through group-perceived job satisfaction at the clinic. Against expectations, social capital was not found to have a direct effect on early failure of fillings. This lack of a direct effect does not detract from our main finding of social capital playing a role in early filling failure through job satisfaction, because the approach to testing mediation does not require (mathematically or theoretically) a significant direct pathway from the predictor to the outcome. Overall, our main finding corroborates previous research, which has found that social capital leads to job satisfaction [8, 11–13, 19] and to better self-reported performance [8, 19].

Social capital was associated with job satisfaction and stress at the clinic level. That is, clinics that had higher levels of social capital had staff with more job satisfaction and less stress. This pathway from social capital to job satisfaction has received consistent support at the individual and at the organizational level [8, 11–13, 19]. Furthermore, consistent with our own findings, there is also support for the influence of social capital on the health of workers [10, 13, 16, 18]. The amount of variance in social capital attributed to the clinical level supports the notion that social capital is an upstream factor that can drive either a positive, motivating path or a negative, health deteriorating path, corresponding to, for example, the Psychosocial Safety Climate [61].

Our finding that high job satisfaction at the clinic level predicts a lower probability of early filling failure (which is an indicator of high care quality) is in accordance with previous organizational level research. For example, a recent review of the Happy Worker Hypothesis at work-unit level using longitudinal design research demonstrated consistent, although scarce, evidence that work-unit wellbeing predicts sales or other economic measures of work-unit performance (although to the best of our knowledge, no studies have addressed the quality of the performed work) [40]. Also corroborated by our findings, a test of the pathway from employee satisfaction to multiple dimensions of performance (e.g., customer satisfaction and worker productivity) using data from home care organisations found that job satisfaction predicted care quality, and that organisations with a high employee satisfaction also had higher levels of customer satisfaction [41]. Interestingly, however, that investigation did not find a clear link from employee satisfaction to productivity. This result can be interpreted as meaning that organizations with high employee satisfaction allow staff to focus on quality performance without the need to increase the sheer amount of work performed. Our findings corroborate the idea that job satisfaction plays a role in providing quality outcomes.

Contrary to our expectations, we did not find support for the proposed pathway from stress at the clinic level to care quality. The same study as referenced above found that the average level of emotional exhaustion was related to reduced customer satisfaction and also to reduced productivity [41]. Meaningfulness and pride in dental work is closely related to delivering high care quality in the clinical encounter with patients [33]. As has been demonstrated in prior research, dentists and dental nurses working in fast-paced environments tend to compromise on ergonomics and administrative tasks rather than lower the quality of treatment [62]. Hence, it could be that worker stress at the clinics in this study did not reach a level that threatened the quality of care in terms of filling longevity. Instead, the dental staff might have compromised on the quality of their own ergonomics or administrative tasks, or perhaps they slowed down the work pace to maintain the quality of their performance in the face of stress.

We included three potential moderating local factors concerning size of filling and placement in the mouth because we expected that the higher risk of failure in general, the more likely it would be to find that work environmental factors affected work results. In a recent 13-year follow-up study on dental filling longevity, survival analyses revealed that the most important factor affecting survival of composite fillings was if the filling was placed in molar versus premolar teeth, and in particular during the first years after it is made [49]. Accordingly, and taking the limited statistical power of our study into consideration, it is not surprising that our findings applied to molar teeth only.

### Implications

Recently there has been much research on social capital, and all indications are that social capital in the workplace is important for predicting employee health and wellbeing [8, 10, 13], work ability [14, 15], use of antidepressant medicine [16], long-term absence due to illness [18], and retention of healthcare staff [11]. However, proposing useful actions based on surveys of psychosocial factors is more challenging than handling physical risk factors at workplaces. Workplace leaders and their relationships with employees are key social factors in the work environment and for implementing organizational policies and strategies. A systematic approach integrating occupational health and safety management with other organizational processes, such as strategic planning, is crucial for success [63]. Previous research has pointed to the importance of managing dentistry in a way that respects the professional ethos of dentists [62], and this importance of social capital is in line with a professional ethos that emphasises trust-based relationships. Furthermore, it has been shown to be possible to intervene to increase social capital through targeted processes [64].

For patients, employees, organizations, and society, it is of great importance that dental fillings are of a quality that ensures their longevity, and that dental healthcare staff thrive in their work. In line with findings from a population study from Finland [49], we found that one out of five fillings in molars was replaced within 3 years of being first made. Every time a filling is replaced, the tooth becomes a little weaker, and filling replacement is also associated with economic, emotional, and physical costs. An increase in workplace social capital leading to a single standard deviation worth of a higher average level of job satisfaction is an achievable goal for organizations and corresponds to the established level of 5–10 points being a minimally important score difference for most COPSOQ scales [65]. Even if the clinical work environment is improved a small amount, that improvement would have the potential for a substantial improvement of public health. Social capital is related to less stress and improved job satisfaction among employees as well as better outcomes for the patients. Therefore, workplace social capital can be a relevant consideration for pursuing the Quadruple Aim Principle [4] in practice.

Theoretically, our findings suggest that the Happy Worker Hypothesis can be extended to the group level, in that workplaces where workers are satisfied with their job produce better quality output.

### Strengths and limitations

The main advantage of the present study is that it combines questionnaire and prospective register data which has the effect of 1) eliminating the risk of common methods bias between exposures and outcomes, and 2) providing an indicator of care quality based on objective criteria rather than subjective staff or patient ratings. The research design furthermore made it possible to integrate additional socioeconomic patient data from Statistics Sweden and thus control for potential confounding factors due to differences in patient characteristics at different clinics.

Another advantage is that the workplace surveys were based on an internationally recognized, validated instrument for risk assessment at workplaces [46]. The international recognition and validation add to the credibility of the measurement and facilitates a transfer of research findings to practical use in the workplace. From the surveys, we included measures aggregated from individual ratings to the clinic level, which provides a more accurate and objective estimate of the levels of social capital in the working environment and of the collective experience of stress and job satisfaction. In this study, the reliability (i.e., ICC2) for the aggregated group means was good for workplace social capital, while slightly below acceptable for job satisfaction and stress (according to criteria suggested by Koo & Li [54]). The amount of variance in the employees’ responses that could be explained by the clinic they work at was the typical maximum seen in applied field research of organisations (p 362 in [51]), and a small to medium effect was seen for stress and job satisfaction (according to the criteria suggested by LeBreton & Senter [53]). In terms of within-group agreement in the workplace, there appeared to be a reaonsable level of consistency in response from employees within a particular workplace for all measures of job satisfaction, stress, and social capital (in accordance with the general rule of thumb for reliability [55]). The ethical permission obtained for the present study did not allow for a study design to include individual-level work environmental factors. The amount of variance left to be explained at individual level, however, makes this relevant to consider in the future.

A limitation is that while early filling failure was measured at three points in time, we only looked at the cross-sectional relationships regarding the working environment factors. For that reason, we are unable to conclusively establish the direction of the pathways among the work environment factors. In addition, the study is based on a relatively small sample of workplaces, which implies a limited statistical power.

In future studies it will be important to replicate this study in different settings and in relation to various working conditions and using different indicators of quality. In particular, it will be relevant to have repeated measures for all variables to be able to better identify causality.

## Conclusion

Healthcare in welfare societies like Sweden has increased in cost over the past 50 years, highlighting the need for developing strategies to simultaneously improve care quality while also minimizing costs. Through this innovative study that uses survival of dental fillings (which are a common and costly form of treatment) as an indication of care quality, we show that strong social capital is associated with reduced stress in dental clinics and can indirectly predict higher quality of care through increased job satisfaction among staff. This conclusion supports the idea that promoting a good work environment is an important strategy to consider when pursuing the combined goal of improved care quality and cost containment.

## Data Availability

The dataset analysed for this study is available from the corresponding author upon reasonable request. A supplementary ethical approval would be needed for the request to be fulfilled.
